# Re-consent practices in biobanks in Japan: current status and stakeholder perspectives

**DOI:** 10.1007/s12687-025-00820-4

**Published:** 2025-07-17

**Authors:** Hiroko Terui-Kohbata, Hiyori Ueda, Masayuki Yoshida

**Affiliations:** 1 Life-Science and Bioethics Research Center, Institute of Science Tokyo, Tokyo, Japan; 2 Department of Life Sciences and Bioethics, Graduate School of Medical and Dental Sciences, Institute of Science Tokyo, 1-5-45 Yushima, Bunkyo, Tokyo, 113-8510 Japan

**Keywords:** Autonomy, Biobank, Genome data-sharing, Pediatric research, Re-consent

## Abstract

Pediatric research in rare diseases relies on sharing biological specimens, clinical data, and analytical information among researchers. Re-consent is essential in longitudinal biobank (BB) research to ensure that pediatric participants remain informed and willing to continue. However, the issue of re-consent—obtaining consent once participants reach adulthood—remains a significant ethical concern. This study examined the current practices of re-consent acquisition in Japanese BBs and explores stakeholder opinions regarding genomic data sharing. A survey of 41 BBs revealed that only 25% of those handling pediatric samples obtained re-consent, all via written informed consent. Although 71% of respondents recognized the necessity of re-consent, the methods used to obtain it varied. Stakeholders identified ethical and logistical challenges, including privacy concerns and administrative burden. Various re-consent methods were suggested, with preferences depending on feasibility and ethical considerations. The findings highlight the need for policy discussions to balance data-sharing benefits with participant rights and privacy protection in pediatric genomic research. Determining optimal re-consent methods requires continued stakeholder engagement, including research participants and the public.

**Supplementary Information:**

The online version contains supplementary material available at 10.1007/s12687-025-00820-4.

## Introduction

The landscape of medical research has evolved significantly due to advances in genetic analysis and information technology (Botkin et al. 2015). Pediatric research increasingly focuses on genetic and environmental factors contributing to diseases, single-gene and multifactorial disorders, and potential treatment and prevention strategies (Rahimzadeh et al. 2018; Hartsock et al. 2019; Patrinos et al. 2022). Given the rarity of many pediatric conditions, securing a sufficient sample size is challenging, necessitating long-term storage and data sharing (Rahimzadeh et al. 2018; Patrinos et al. 2022). However, the long-term retention and dissemination of pediatric data pose ethical challenges, including privacy concerns, the potential for data misuse, and risks of genetic discrimination (Rahimzadeh et al. 2018; Patrinos et al. 2022). Thus, striking a balance between the benefits and risks of genomic data sharing is imperative (Patrinos et al. 2022).

Biobanks (BBs) have been developed globally as research platforms, collecting and storing biological samples, clinical data, and analysis data from patients and healthy individuals. These resources are shared with researchers to identify disease causes and develop treatments and prevention methods. In Japan, several BBs have been established, classified into types such as resident participation - disease-oriented, healthy population - patient population, single-site - multisite, medical institution - cohort, and treatment - research. The three major BBs in Japan are Biobank Japan (BBJ, ~ 267,000 cases), a disease-oriented BB; National Center Biobank Network (NCBN, ~ 139,000 cases), a BB at a medical institution; and Tohoku Medical Megabank (TMM, ~ 151,000 cases), a resident participation cohort BB. Other BBs at research institutes and medical institutions also collect and utilize samples and data. In the United States (U.S.), BBs exclusively enroll adult participants, with only 40% including any children and 2% focused entirely on pediatric patients (Henderson et al. 2013). Similarly in Japan, most BBs recruit adult subjects, with some, like BBs at children’s hospitals and resident cohort BBs, targeting pediatric subjects. Statistical data on age at time of sample collection from nine biobanks, including the three largest in Japan, reveals that children and adolescents are extremely rare (Biobank Network Japan 2025). However, recent developments suggest an increase in pediatric biobanking efforts, such as those reported by Brothers et al. (2019), who highlight expanded enrollment of pediatric patients in genomic research programs. Pediatric biobanking presents new opportunities to conduct valuable translational research that can benefit child populations. However, the involvement of minors—who are often considered incapable of providing fully informed consent—raises numerous ethical, legal, and social challenges. Moreover, most national and international guidelines on biobanking are non-binding, and discrepancies across documents can result in inconsistent interpretations. As a result, stakeholders may receive only limited guidance on how to ethically manage pediatric participation in biobanking initiatives (Prince et al. 2024).

The “Ethical Guidelines for Life Science and Medical Research” in Japan require informed consent (IC) from individuals aged 16 and above, while minors require parental consent with informed assent. The necessity for the research participant to provide re-consent upon reaching adulthood remains debated, as ethical guidelines do not explicitly require it when the research continues within the scope of the initial broad consent given by the parents (Ministry of Education, Culture, Sports et al. 2021). The term “initial broad consent” refers to a type of informed consent allowing for future unspecified use of biological materials and data within a defined governance framework. In Japan, broad consent is accepted under the Ethical Guideline for Life Science and Medical Research, and more the guidelines allow the use of samples and data based on an opt-out approach after obtaining broad consent. As Garrison et al. (2016) note, individual attitudes toward broad consent can be context-dependent and influenced by cultural norms.

This study investigates the current status of re-consent acquisition in Japanese BBs and gathers stakeholder opinions through a structured survey. Under the Ethical Guideline for Life Science and Medical Research Involving Human Subjects in Japan, there is no “exemption” from informed consent, even for minimal-risk research. Consequently, opt-out procedures have become a de facto standard, particularly in observational studies and secondary use of data and biospecimens. In designing the questionnaire on re-consent, both opt-in and opt-out models were included to align with permissible practices under the guidelines and to examine their cultural acceptability. These approaches remain a subject of ongoing debate in Japan, reflecting concerns about participant burden, operational feasibility, and varying interpretations of autonomy.

## Materials and methods

### Subjects

The survey targeted 41 cohort BBs or medical-institution-attached BBs, excluding depository BBs, listed on the Japan Agency for Medical Research and Development’s (AMED) BB Information List. Depository biobanks were excluded because they typically store samples without direct interaction with participants, making the assessment of re-consent procedures inapplicable.

### Questionnaire

Informed consent was obtained from all individual participants included in the survey. Participants completed the survey either online or in paper format. The survey was conducted from January 25 to March 15, 2023, and approved by the Research Ethics Committees (Approval Number: C2022-042).

The questionnaire covered the following (see Supplementary File 1 for full questionnaire):

1. Current re-consent practices (whether re-consent is obtained and how).
2. Opinions on re-consent (its necessity, preferred methods, and perceived benefits/risks of genomic data sharing).
3. BB attributes (type, pediatric sample handling, genomic data management).
4. Respondent attributes (role in BB, occupation).

### Statistical analysis

The data were analyzed using simple aggregation and cross-tabulation. Chi-square tests and residual analysis were applied where relevant, with significance set at *p* < 0.05.

## Results

### Respondent characteristics

A total of 21 BBs (51.2%) responded. Among them, 12 BBs (57.1%) handled pediatric samples, and 3 BBs (14.3%) distributed genomic data. Nine respondents were BB managers, while ten were BB coordinators.

### Current re-consent practices

Among the 12 BBs handling pediatric samples, only 3 BBs (25.0%) had obtained re-consent, all using face-to-face written consent.

### Attitudes toward re-consent

A majority of respondents (71.4%) supported the necessity of re-consent, though opinions varied regarding the most appropriate method. The suggested approaches included:

- Opt-out via email/post notification (*n* = 4).
- Written informed consent (*n* = 3).
- Opt-in via email/post notification (*n* = 2).
- Opt-out via website notice (*n* = 2).
- Method-dependent on case-specific factors (*n* = 3).

The preference for opt-out with individual notice was based on its practicality, with respondents noting that while obtaining informed consent (IC) is ideal, it is often challenging to implement. Supporters of written consent emphasized the value of face-to-face explanations, as they help confirm the participants’ intentions and provide an opportunity to address any questions or concerns. Opt-out with public notice was chosen to reduce the administrative burden on BB staff, whereas opt-in with individual notice was favored for its ability to clearly confirm participants’ willingness to continue their involvement in a realistic and reasonable manner.

### Attitudes toward the pros and cons of sharing pediatric genomic data

**Fig. 1:**
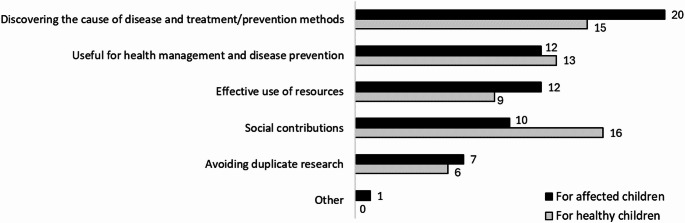
Benefits of genomic data sharing for pediatric subjects and their parents. The Black and white bars indicate the benefits for the affected and healthy children, respectively. The horizontal axis indicates the number of participants. Multiple selections were permitted. * indicates a significant difference at the 5% level

Participants were asked, “What are the benefits for affected and healthy children or their parents in sharing genomic data?” and were asked to select the top three items from six choices. Figure 1 shows the results. For affected children, the most cited benefit was “discovering disease causes and treatment/prevention methods” (95.2%), significantly higher than for healthy children (71.4%, *p* < 0.05). For healthy children, “social contributions” (76.2%) was the most recognized benefit.

**Fig. 2:**
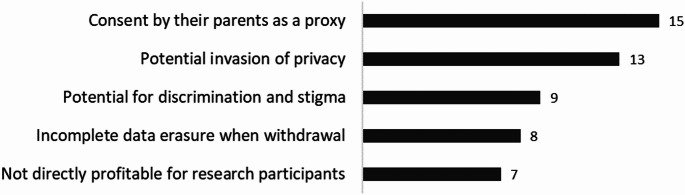
Issues of sharing pediatric genomic data. The horizontal axis represents the number of respondents. Multiple selections were permitted

Participants were further asked, “What are the issues that arise when sharing pediatric genomic data?” and were requested to select the top three of six choices. Figure 2 shows the results. Key concerns included “parental consent in place of the child” (71.4%) and “potential privacy invasion” (61.9%). Genetic discrimination and stigma were cited by over 40% of respondents.

## Discussion

### Ethical considerations and practical challenges in re-consent

Approximately 70% of BB stakeholders view re-consent as necessary. This aligns with findings from international studies in which 73% of experts involved in human research protection supported re-consent for genetic research (Lemke et al. 2010). While re-consent respects participant autonomy (Wallace et al. 2016), challenges include the logistical burden and the potential discomfort of re-engaging participants.

Studies in Canada and the UK show that research participants feel more engaged when asked for re-consent (Master et al. 2013; van der Velden et al. 2023). However, concerns exist regarding administrative feasibility, costs, and participant burden. In this study, only 25% of BBs implemented re-consent, indicating significant challenges in its practical application.

Moreover, requiring re-consent at the age of consent can serve as a mechanism to promote public trust in research institutions. When individuals are actively given the opportunity to decide on the continued use of their samples and data, it enhances their sense of agency and may contribute to greater societal acceptance of biobanking practices. This aligns with discussions on dynamic consent models that emphasize transparency and ongoing participant engagement (Kaye et al. 2015).

In Japan, the continued use of samples and data in pediatric genomic research based on broad consent given by parents is commonly accepted, in accordance with guidance provided by national ethical guidelines. This acceptance typically applies only to biospecimens and information that were already collected prior to the child reaching adulthood. When such data or samples are used in ongoing or new research, it is customary to notify the public through the disclosure of study information and to provide an opportunity for opt-out, rather than seeking renewed individual consent. Cultural norms that emphasize family decision-making may support such practices, yet this approach may conflict with emerging standards of individual autonomy and informed consent.

Further illustrating these challenges, a semi-structured interview survey of 30 Australian young adults (18–34 years old) who were former pediatric oncology patients and had stored biospecimens revealed that 60% believed biobanking permission should be re-obtained in adulthood, yet 70% were unaware of their previously banked tissue (Rush et al. 2015). From a researcher’s perspective, arguments against re-consent include the impracticality of obtaining individual consent, high resource demands, and the concern that re-consent requests might cause undue distress to participants (Wallace et al. 2016). Given that only one-quarter of the BBs in this study had implemented re-consent, its widespread adoption remains a significant challenge.

### Re-consent methods: finding a feasible approach

Various alternative approaches to re-consent, such as opt-out notification and complete anonymization, have been proposed. However, each method presents challenges. Opt-out approaches raise concerns about the effectiveness of information dissemination, as previous studies suggest that participants strongly prefer explicit permission for the use of their genetic data (Ludman et al. 2010). On the other hand, complete anonymization removes the need for re-consent but also limits participants’ rights, such as withdrawing consent or receiving research results. Given Japan’s legal framework, anonymization alone may not fully meet ethical compliance requirements. In Japan, genomic data are classified as personal information under the Act on the Protection of Personal Information. the Ethical Guideline for Life Science and Medical Research Involving Human Subjects in Japan (2021) further emphasize the importance of data protection and the safeguarding of individual rights, even in research involving anonymized data. Consequently, complete anonymization does not necessarily exempt a study from ethical oversight. Comparative frameworks, such as the EU’s GDPR, highlight similar concerns, where pseudonymization still requires legal safeguards (Shabani and Borry 2018).

BB stakeholders emphasized that the most appropriate re-consent method depends on several factors, including the management status of samples and data, accessibility to participants, timing of re-consent, and the operational burden on BBs. To determine the most suitable approach, it is crucial to conduct surveys involving research participants, their proxies, and the public while continuously evaluating the necessity and feasibility of re-consent practices in Japan. Digital tools, such as secure web portals and mobile apps, are useful for promoting communication with participants and enabling re-consent and dynamic consent (Gesualdo et al. 2021; Lee et al. 2024). While these approaches have been adopted internationally, they have not yet been implemented in Japanese biobanks and remain a future direction.

### Balancing data sharing benefits and privacy risks

Many respondents in this survey highlighted “discovering the causes of diseases and developing treatment/prevention methods” as key benefits of sharing pediatric genomic data. Similarly, a U.S. study involving 163 parents of infants found that those who participated in a clinical trial prioritized the “direct benefit for the child” significantly more than non-participating parents (Shah et al. 2018), a trend supported by multiple studies (Hoberman et al. 2013; Nathe et al. 2023). Some ethical concerns raised by stakeholders—particularly participants’ expectations of personal benefit—may reflect a phenomenon known as the ‘therapeutic misconception.’ This refers to the mistaken belief that participating in research will provide direct therapeutic benefits, even though the primary objective of research is to generate generalizable knowledge rather than to deliver clinical care (Appelbaum et al. 1987). In pediatric biobanking, such misconceptions may influence parental decision-making, especially when consent is motivated by hopes of discovering a cure or advancing treatments for their child. Moreover, altruism has been identified as the second most common motivation for participating in research after direct personal benefit (Nathe et al. 2023). Therefore, it is essential to clearly communicate that participation in research primarily contributes to broader societal benefits—such as the development of future treatments and preventive strategies for other children—rather than immediate clinical outcomes for the participant. Conversely, concerns about privacy and genetic discrimination were prominent. Over half of the respondents cited “the possibility of invasion of privacy,” while more than 40% mentioned risks of “discrimination and stigma.” A survey of 10,731 Japanese individuals, including cancer patients and their families, indicated that those with a personal or family history of disease were more concerned about genetic discrimination than healthy individuals (Ri et al. 2023). Another study of 1,760 parents of newborns who declined genomic research participation found that privacy and discrimination concerns influenced their decision (Genetti et al. 2019).

Genetic discrimination remains a global issue, with policies differing by country in terms of insurance enrollment, employment, and public awareness (Kim et al. 2021). In North America, concerns differ by race, nationality, and insurance systems (Hall et al. 2005; Prince & Marks 2021), whereas seven Asian countries, including Japan, report lower concerns in health insurance due to universal coverage (Kim et al. 2021). However, in Japan, younger generations are increasingly worried about genetic discrimination in employment, while concerns about marriage and pregnancy discrimination remain significant (Hishiyama et al. 2019). These findings suggest that in Japan, genomic research participation concerns extend beyond medical risks to social and economic implications. To mitigate these risks, measures should be implemented to prevent discrimination and enhance public literacy on genomic research.

Advances in genomic analysis have improved accuracy and expanded clinical applications. The American College of Medical Genetics and Genomics (ACMG) has identified actionable genes for clinical disclosure, including secondary findings. In Japan, the Tohoku Medical Megabank pilot study returned results on pathogenic variants for familial hypercholesterolemia, Lynch syndrome, and hereditary breast and ovarian cancers (Kawame et al. 2022; Ohneda et al. 2023). While genomic information can facilitate health management, it may also heighten anxiety and concerns about unexpected hereditary disease diagnoses. To ensure responsible data sharing, genetic experts should provide appropriate information and psychosocial support. In practice, re-consent is not only relevant at age thresholds but also when returning individual results with clinical significance. Such key decision points warrant renewed consent to ensure ongoing voluntary participation (Appelbaum et al. 2014). Given that many participants initially enrolled through parental consent, re-consent becomes increasingly vital in allowing individuals to make informed decisions about their genetic data. Genetic counselors can play a crucial role in re-consent processes by supporting participants in understanding genomic results and making informed decisions. Their involvement may enhance participant autonomy and reduce anxiety about unexpected findings.

### Study limitations

Since the respondents of this study were just 21 BB stakeholders (response rate was approximately 50%), the results did not reflect all BBs in Japan. However, this study was a first step in the discussion on the pediatric genome data sharing among nationwide Japan BBs.

## Conclusion

Promoting data sharing while protecting participants’ rights is essential to maximizing genomic research benefits. Determining optimal re-consent methods requires continued stakeholder engagement, including research participants and the public. Simultaneously, robust safety measures and legal protections must be established. The recent enactment of Japan’s genomic medicine law in May 2023 signifies an important step toward addressing these ethical challenges. While this legislation functions as a basic, non-binding framework law and does not impose legal sanctions, it may nonetheless influence how biobanks implement re-consent procedures and manage data sharing. At present, specific implementation measures are being developed under the Basic Plan.

## Electronic supplementary material

Below is the link to the electronic supplementary material.

Supplementary Material 1

## Data Availability

No datasets were generated or analysed during the current study.
