# Supplementary material for: Re-consent practices in biobanks in Japan: current status and stakeholder perspectives
Source: J Community Genet. 2025 Jul 17;16(5):639–45. doi: 10.1007/s12687-025-00820-4 (PMC12401817; doi:10.1007/s12687-025-00820-4)
Supplement: Supplementary file 1 — Supplementary Material 1 [file 12687_2025_820_MOESM1_ESM.pdf]

Questionnaire (Biobank version)

I. Biobank Attributes

1. Which of the following best describes your biobank? (Multiple answers possible)

⑴ Population-based Biobank

⑵ Disease Biobank

⑶ On-demand biobanks (biobanks that prospectively collect biological samples at the request of researchers)

⑷ Others (please specify): ___________

2. Who is obtaining consent for your biobank? (Multiple answers possible)

⑴ Doctor in charge

⑵ Biobank staff (including research assistants, CRCs, etc.)

⑶ Others (please specify): ___________

3. Please tell us the organizations/people to whom your biobank can provide samples/data. (Multiple answers possible)

⑴ Domestic commercial organizations

⑵ Domestic academic/Non-profit organizations

⑶ Foreign commercial organisations

⑷ Foreign academic/Non-profit organizations

4. Does your biobank handle samples from children (those under 15 years old)?

⑴ Yes → Number of samples (   )

⑵ No

5. Does your biobank provide genome data?

⑴ Yes → Number of cases (   )

⑵ No

***For biobanks that do Not handle pediatric samples or genome data, please go to page 5.**

Please tell us how your biobank handles cases where donors are under 15 years old.

II. Informed consent (IC)

IC refers to the consent of research subjects, etc. to the implementation or continuation of research (including the handling of samples and data), which is given voluntarily after receiving sufficient explanations from researchers, etc. or those who only provide existing samples and data about the purpose, significance, and methods of the research, the burden on the research subjects, the expected results (including risks and benefits), etc. (From the Ethical Guidelines for Life Science and Medical Research Involving Human Subjects, March 23, 2021 (partially revised March 10, 2022))

6. Do you provide the following information to the donor parents during the IC process? Please circle the number of the item that you provide. (Multiple answers allowed)
***Please answer based on the most recent IC document.**

(1) Scope of use of provided samples/data
(2) In the case of rare diseases, there is a possibility that individuals may be identified even if they are anonymized
(3) Regarding the infringement of the right to self-determination through broad consent
(4) Regarding the infringement of privacy through the provision of genome data and information leaks
(5) Potential issues such as discrimination and stigma
(6) Regarding the need for donor's re-consent
(7) Possibility that a hereditary genetic disorder may be discovered as a secondary finding
(8) Regarding the impact on blood relatives of disclosure of individual genetic analysis results

III. Informed Ascent (IA)

IA refers to a research subject who is objectively judged to lack the ability to provide IC being given an explanation of the research to be conducted or continued in easy-to-understand terms according to his/her level of comprehension, understanding that the research will be conducted or continued, and expressing his/her consent. (From the Ethical Guidelines for Life Science and Medical Research Involving Human Subjects, March 23, 2021 (partially revised March 10, 2022))

7. At what age did you obtain informed assent?

(1) Preschool
(2) Elementary school
(3) Upper elementary school
(4) Middle school and above
(5) Regardless of age (maturity, etc.)

8. What information is provided to doNors in the informed assent process? Please circle all that apply.

Please answer based on the latest IA document.

	**Elementary school**	**Upper elementary school**	**Middle school and above**
Regarding organizations/individuals that may provide collected samples and data			
Regarding genetic analysis			
	**Elementary school**	**Upper elementary school**	**Middle school and above**
Scope/purpose of research using collected samples and information			
Regarding the infringement of privacy through the provision of genome data and information leaks			
Possibility of secondary findings and their disclosure			
Regarding the need for re-consent			
What to do if re-consent is not obtained			
How to withdraw (contact details, website, etc.)			

IV. Handling of pediatric samples/data

9. Please tell us the form and recipient of child specimen/genome data for genetic analysis research.

(○: Provided, ×: Not provided, △: Conditional)

	**Condominiums**	**Provided through joint research**
Domestic commercial organizations		
Domestic academic/non-profit organizations		
Foreign commercial organizations		
Foreign academic/non-profit organizations		

10. Does the answer differ from how adult samples/genome data is handled?

(1) Yes → What are the specific differences? ( )

(2) No

V. Re-consent

11. Have you obtained re-consent from individuals who provided samples/data during childhood?

(1) Yes

(2) No (*please proceed to Question 18*)

12. At what age did you obtain re-consent?

(1) 16 years old (including 15 years old who have graduated from junior high school)

⑵ 17 years old

⑶ 18 years or older

⑷ Others (please specify): ___________

13. How do you obtain re-consent? (Multiple answers possible)

⑴ Obtain written consent in person

⑵ Send the document and get the consent form returned

⑶ Send an email and get a response

⑷ Notify individuals by letter/e-mail and ask them to reply/return it only if they refuse (Passive opt-out style notification)

14. What is the rate at which re-consent is obtained?

⑴ Over 80%

⑵ 60~80%

⑶ About 50%

⑷ 20~40%

⑸ Less than 20%

15. What do you think is the reason for the achievement rate you answered in the question 14? (Multiple answers allowed)

⑴ Cost

⑵ Time

⑶ Human Resources

⑷ Availability of donor contact information

⑸ Provider interest

⑹ Others (please specify): ___________

16. What do you do if re-consent can Not or has Not been obtained?

⑴ Continue to use samples/data

⑵ Continued use of samples/data under certain conditions

⑶ Discard the samples/data

⑷ Others (please specify): ___________

16-1. For those who selected ⑵ in Question 16, please tell us more about the conditions.

( )

Please tell us your thoughts about what to do <u>if the donor is a child (under 15 years old)</u>.

VI. Explanation

17. What do parents, who act as legal representatives, need to understand when obtaining informed consent (IC; consent from a legal representative) for data sharing of pediatric samples? Please rank the following items in order of importance from 1st to 3rd in order of importance.

1st place (   ), 2nd place (   ), 3rd place (   )

(1) Scope of use of provided samples/data
(2) In the case of rare diseases, there is a possibility that individuals may be identified even if they are anonymized
(3) Regarding the infringement of the right to self-determination through broad consent
(4) Regarding the infringement of privacy through the provision of genome data and information leaks
(5) Potential issues such as discrimination and stigma
(6) Regarding the need for re-consent of donors when they reach the age of consent
(7) Possibility that a hereditary genetic disorder may be discovered as a secondary finding
(8) Regarding the impact on blood relatives of disclosure of individual genetic analysis results

18. What do you think is the information that needs to be communicated to the individual in the informed assent (IA; agreement from a child participant)? Please circle the one that applies.

	**Elementary school**	**Upper elementary school**	**Middle school and above**
Regarding organizations/individuals that may provide collected samples and data			
Regarding genetic analysis			
Scope/purpose of research using collected samples and data			
Regarding the infringement of privacy through the provision of genome data and information leaks			
Possibility of secondary findings and their disclosure			
Regarding the need for re-consent			
What to do if re-consent is not obtained			
How to withdraw (contact details, website, etc.)			

19. How do you feel about the age for obtaining an informed assent compared to the current age at your facility?

(1) Faster is better
(2) Present status is better
(3) Slower is better
(4) It is better to adapt to the individual's level of understanding, without setting an age limit.
(5) others (   )

VII. Re-consent

20. If a donor reaches the legal age for consent, do you think it is necessary to obtain re-consent from individuals who provided samples/data during childhood?

(1) Yes

(2) No → Please proceed to Question 26

21. At what age do you think it would be best to obtain re-consent?

( ) years old

22. How do you think it would be best to obtain re-consent?

(1) Obtaining document consent in person

(2) Obtaining a verbal consent by phone, etc.

(3) Notify by email/mail and have participants return the documents

(4) Notify by email/mail and have the item returned only if rejected

(5) Post it on a website etc. and ask people to contact you if they refuse

(6) Others (please specify): ___________

23. Please explain the reason for your answer to the Question 22.

( )

24. If re-consent cannot be obtained/is not possible, do you think opting out is necessary?

(1) Yes

(2) No → Why is that? ( )

VIII. Data Sharing

25. What form and recipient of samples/genome data from children for genetic analysis research should be provided? Do you think it is appropriate to provide it?

○: Provide it, ×: Do Not provide it, △: Conditional

	**Condominiums**	**Provided for collaborative research only**
Domestic commercial organizations		
Domestic academic/non-profit organizations		
Foreign commercial organisations		
Overseas academic/non-profit organizations		

26. How do you think consent should be obtained for individual studies using children's genome data held by your biobank?

(1) Used after obtaining individual consent

(2) The doNor or surrogate chooses the research they wish to be involved in.

(3) Because broad consent was obtained, samples/data will be used in multiple studies without individual consent

(4) Others (please specify): ___________

27. What do you think are the benefits to the patient or the legal representative of sharing genome data of the "child patient"? Please circle your top three answers.

(1) Discover the causes, treatments, and prevention methods

(2) Avoid duplicate research

(3) Resources can be used more effectively (including reducing the burden on the individual)

(4) It can be used to help manage health and prevent disease.

(5) Contributing to society

(6) Others (please specify): ___________

28. What do you think are the benefits to children or their legal representatives from sharing genome data of "healthy children"? Please circle your top three answers.

(1) Discover the causes, treatments, and prevention methods

(2) Avoid duplicate research

(3) Resources can be used more effectively (including reducing the burden on the individual)

(4) It can be used to help manage health and prevent disease.

(5) Contributing to society

(6) Others (please specify): ___________

29. What do you think are the biggest challenges to sharing pediatric genome data? Please circle your top three.

(1) The consent is given by a parent rather than by the donor himself

(2) Potential for privacy violations

(3) It may Not be in the individual's direct interest

(4) Potential for discrimination and stigma

(5) The fact that you canNot completely cancel your data even if you withdraw your consent

(6) Others (please specify): ___________

IX. Respondent attributes

30. What is your role at the biobank? (multiple answers possible)

(1) Biobank Director

(2) Sample Management

⑶ informed consent (IC; consent from a legal representative) acquisition
⑷ Ethics Officer
⑸ Coordination with clinicians
⑹ Coordination with researchers
⑺ Administrative staff
⑻ Others (please specify): ___________

31. What is your job? (multiple answers possible)

⑴ Doctor
⑵ Nurse
⑶ Clinical Laboratory Technologist
⑷ Certified Genetic Counselor
⑸ Clinical Research Coordinator (CRC)
⑹ Ph.D.
⑺ Researcher
⑻ Others (please specify): ___________

This concludes the questionnaire. Thank you for your response.
